# High M2-TAM Infiltration and STAT3/NF-κB Signaling Pathway as a Predictive Factor for Tumor Progression and Death in Cervical Cancer

**DOI:** 10.3390/cancers16142496

**Published:** 2024-07-09

**Authors:** George Alexandre Lira, Fábio Medeiros de Azevedo, Ingrid Gabrielle dos Santos Lins, Isabelle de Lima Marques, Giovanna Afonso Lira, Christina Eich, Raimundo Fernandes de Araujo Junior

**Affiliations:** 1 Cancer and Inflammation Research Laboratory, Department of Morphology, Federal University of Rio Grande do Norte Natal, Natal 59072-970, RN, Brazil; dr.georgelira@gmail.com; 2 Postgraduate Program in Health Science, Federal University of Rio Grande do Norte, Natal 59072-970, RN, Brazil; isalima.24@hotmail.com; 3 Department of Radiology, Leiden University Medical Center, 2333 ZA Leiden, The Netherlands; c.eich@lumc.nl; 4 League Against Cancer from Rio Grande do Norte, Advanced Oncology Center, Natal 59075-740, RN, Brazil; ggaby_gabrielle@hotmail.com (I.G.d.S.L.); giovannaalira@gmail.com (G.A.L.); 5 Pathology Department, Federal University of Rio Grande do Norte, Natal 59012-570, RN, Brazil; fabiopathuol@gmail.com; 6 Postgraduate Program in Functional and Structural Biology, Federal University of Rio Grande do Norte, Natal 59072-970, RN, Brazil

**Keywords:** M2-TAM, STAT3, cervical cancer, recurrence, overall survival, tumor progression

## Abstract

**Simple Summary:**

Once in an immunosuppressive tumoral microenvironment (TME), macrophages take over an essential role by activating signaling pathways such as STAT3 and NFkB to keep the immune suppression and tumor progression. However, this role in cancer cervical (CC) is not still clear. For that, we studied the interaction of tumor associated macrophages (TAM) and STAT3 and NFkB pathways with of several cancer hallmarks in 691 patients with CC, and more importantly, their impact on clinical outcome of these patients. Interestingly, a strong immunosuppressive loop was identified via TAM and STAT3 and NFkB pathways in TME which reverberated in a higher expression of tumor progression markers, and consequently in a poor clinical outcome.

**Abstract:**

Introduction: The tumor microenvironment (TME) plays a crucial role in the progression, invasion, and metastasis of cervical carcinoma (CC). Tumor-associated macrophages (TAMs) are significant components of the CC TME, but studies on their correlation with CC progression are still controversial. This study aimed to investigate the relationship between TAM infiltration, the STAT3/NF-κB signaling pathway, and Overall Survival (OS) in CC patients. Methods: In a retrospective study, 691 CC patients who had received a definitive histopathologic diagnosis of CC scored by the FIGO staging system and not undergone preoperative treatment were selected from a database. The effect of TAM infiltration on tumor progression biomarkers using Tissue Microarray (TMA) and immunohistochemistry was evaluated. Furthermore, the impact of the expression of these biomarkers and clinical–pathological parameters on recurrence-free (RF) and OS using Kaplan–Meier and multivariable Cox regression methods was also analyzed. Results: High stromal CD163 + 204 + TAMs density and via STAT3 and NF-κB pathways was relevant to the expression of E-cadherin, Vimentin, MMP9, VEGFα, Bcl-2, Ki-67, CD25, MIF, FOXP3, and IL-17 (all *p* < 0.0001). In addition, elevated TNM staging IV had a strong association correlation with STAT3 and NF-κB pathways (*p* < 0.0001), CD25 (*p* < 0.001), VEGFα (*p* < 0.001), MIF (*p* < 0.0001), and Ki-67 (*p* < 0.0001). On the other hand, overall and recurrence survival was shown to be strongly influenced by the expression of SNAIL (HR = 1.52), E-cadherin (HR = 1.78), and Ki-67 (HR = 1.44). Conclusion: M2-TAM and via STAT3/NF-κB pathways had a strong effect on CC tumor progression which reverberated in the severity of clinicopathological findings, becoming an important factor of poor prognosis.

## 1. Introduction

Cervical cancer is an important public health problem with a worldwide impact, especially in underdeveloped countries, with high incidence and mortality rates [1,2,3]. Patients with cervical cancer have a rate of 5-year relative survival of 91% when the cancer is diagnosed at earlier stages, dropping to 60% when diagnosed after it spreads to nearby tissues, organs, or regional lymph nodes [4].

Prognostic factors for cervical cancer include race, age, stage, grade, histologic type, tumor volume, lymph node involvement, performance status, and the treatment received [5]. However, new studies are needed on protein biomarkers for cervical cancer prognosis that can guide the treatment. In this line, immunological indicators have been considered important prognostic factors for cervical cancer [6]. Tumor-associated macrophages (TAMs) comprise the largest number of immune cells and the more important part of the tumor microenvironment (TME), acting as immune and tumor regulators [7]. In this context, they have been identified as prognostic factors and potential targets for immunotherapy [7,8,9].

Within tumors by interacting with tumor cells, macrophages play a key role in regulating the immune response, promoting the tumor escape to immunosurveillance [10]. When they shift towards the CD163 + 204 + M2-TAM phenotype through the activation of the signal transducer activator (STAT3) pathway caused by an immunosuppressive cytokine loop, they promote immunosuppression, drug resistance, tumor progression, and metastasis [11,12]. Tumors release immunosuppressive cytokines that stimulate the switch to M2-TAMs via STAT3 in the TME, leading to worse survival in solid tumors [13,14]. Some evidence points out that M2-TAM infiltration has been associated with more advanced FIGO stage and lymph node metastasis [15,16], as well as with higher expression of signal transducer activator (STAT3) in various solid tumors, including breast, prostate, colorectal, melanoma, and brain cancer [17,18]. Reports describe that IL-10, Programmed Death-Ligand 1 (PD-L1), and Transforming Growth Factor beta (TGFβ) promote the STAT3 and Factor Nuclear kappa B (NF-κB) activation which are critical to macrophage polarization [19,20,21]. Furthermore, the STAT3 pathway stimulates specific transcription factors in tumor cells that support the EMT process, resistance to apoptosis, invasion, proliferation, and immunosuppression [17,19,21].

Although there is biological evidence, the clinical impact of M2-TAM’s pro-tumor activity and via STAT3 and NF-κB signaling pathways on the overall survival of cervical cancer patients has not been clarified. Therefore, we conducted a study to validate the association of M2-TAM and via STAT/NF-κB pathways with biomarkers of tumor progression and survival in cervical cancer.

## 2. Material and Methods

### 2.1. Tissue Samples and Data Collection

**Figure 1:**
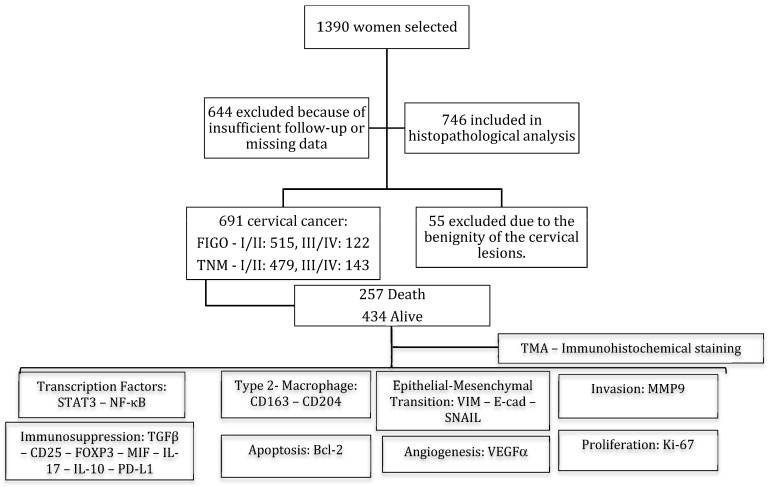
Flowchart of the women included in the study (2003–2017). FIGO/TNM predicts the outcome and determines the type of treatment that will be given. FIGO/TNM: stage I—confined to the organ of origin; stage II—invasion of surrounding organs or tissue; stage III—spread to distant lymph nodes or tissue within the pelvis; stage IV—distant metastasis. Tissue microarray (TMA) immunohistochemistry staining was evaluated for transcription factors (STAT3 and NF-κB), Type 2 Macrophage (CD163-CD204), Epithelial–Mesenchymal Transition (VIM, E-cad and SNAIL), Invasion (MMP9), Immunosuppression (TGFβ, CD25, FOXP3, MIF, IL-17, IL-10, and PD-L1), Apoptosis (Bcl-2), Angiogenesis (VEGFα), and Proliferation (Ki-67).

This research is a retrospective and longitudinal study of 1390 women diagnosed with cervical cancer from 2003 to 2017 registered at the archives of the Advanced Oncology Center at the League Against Cancer from Rio Grande do Norte (LRNCC), Natal, Brazil (Supplementary Materials). After the exclusion of 699 patients (Figure 1), the inclusion criteria adopted to select 691 cases was as follows: (I) patients who underwent surgery with definitive histopathologic diagnosis of cervical cancer, (II) scored by TNM and the International Federation of Gynecology and Obstetrics (FIGO) staging system of cervical cancer [22], and (III) with enough surgical material to perform the tissue microarray (TMA). Furthermore, sociodemographic and clinicopathological data were also collected (Supplementary Materials). The Institutional Review Board (IRB) from LRNCC (CAE 2077017-2644266-2761333-3071847, Natal, Brazil) approved this study. The ethics committee permitted the research, including the collection of information from medical records, as well as paraffin block samples from 691 patients.

### 2.2. Tissue Microarray (TMA) Block Construction and Staining by Hematoxylin and Eosin (HE) and Immunohistochemistry (IHC)

**Figure 2:**
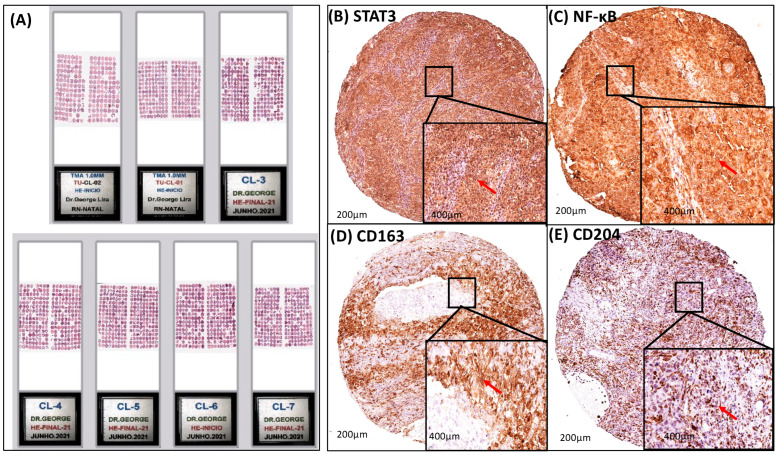
Intracellular signaling modulation in TME by STAT3 and NF-κB pathways via CD163/CD204 M2-TAM. Modulation of intracellular signaling in the TME through the STAT3 and NF-κB pathways and immunostaining of M2-TAM through the biomarkers CD163 and CD204. (**A**) Tissue Microarray (TMA) slides in hematoxylin and eosin (H&E) with duplicates of the 746 patients performed in 2021, (**B**) brownish-brown diffuse staining with strong positive cytoplasmic/nuclear staining for anti-STAT3 (red arrow), (**C**) specific brownish-brown staining with strong positive cytoplasmic staining for anti-NF-κB (red arrow), (**D**) specific moderately diffuse brownish-brown staining with strong positive cytoplasmic staining for anti-CD163 (red arrow), and (**E**) moderately diffuse brownish-brown staining with strong positive cytoplasmic/nuclear staining for anti-CD204 (red arrow) in primary cervical cancer. The immunohistological profile presents images at 200× magnification; 400× magnification is shown in the lower right corner of each image. Arrow: the tumor cell with intracellular staining (cytoplasmic and/or nuclear) depending on each immunohistochemical marker.

The Tissue Microarray blocks were constructed as described by Araujo Jr et al., 2015 [23]. Briefly, all hematoxylin and eosin slides were re-examined by a second experienced pathologist, and two morphologic representative fields of invasive cervical cancer were chosen and encircled with a marker pen. Two samples from each neoplasm, obtained from biopsies and/or surgical specimens, with one millimeter in diameter each, were taken from the donor blocks and transferred to the recipient block, using the Manual Tissue Microarrayer device I (Beecher Instruments, Silver Spring, MD, USA). Three-micrometer cuts of the microarray block were made using the Paraffin Tape-transfer System (Instrumedics, Saint Louis, MO, USA). One of the sections was stained with H&E to confirm the presence of tumor by light microscopy. Immunohistochemical reactions were performed on two different slides to increase the reliability of the results. The histological sections were placed on 8% salinized glass slides for the immunohistochemical method (Figure 2).

#### Immunohistochemistry (IHC)

Immunohistochemistry of TMA slides was performed as described by Araújo Jr et al., 2016 [24]. Using a microtome, 4 µm thin tissue slices were cut from tumors and transferred to gelatine-coated slides. The paraffin-embedded tumor tissue sections were incubated overnight at 4 °C with anti-CD25 to M2-TAM, anti-CD163 to M2-TAM, anti-IL-10, anti-TGF β, anti-VEGF α, anti-IL-17, anti-NFκB, anti-PDL-1, anti-E-Cadherin, anti-BCL-2, anti-MMP-9, anti-Ki-67, anti-Vimentin, anti-CD204, anti-SNAIL, anti-MIF, anti-STAT3, and anti-FOXP3 (Table S1). Slices were rinsed in PBS and treated with a secondary antibody conjugated to streptavidin/Haptoglobin-Related Protein (HRP) (Biocare Medical, Concord, CA, USA). Immunoreactivity to various proteins was visualized using a colorimetric-based detection kit (TrekAvidin-HRP Label + Kit from Biocare Medical, Pacheco, CA, USA) according to the manufacturer’s procedure. High-resolution images of the IHC staining were obtained from scanning an Aperio AT2 slide scanner (Aperio, Vista, CA, USA) at 200× and 400× magnification. The digital images were captured using a high-power objective (200× and 400×) for ImageScope software (12.4.6).

### 2.3. Quantitative Evaluation of Immunostaining

According to the staining intensity, it was considered positive, cells stained in brown in the nucleus, and/or cytoplasm, and/or membrane (Table S2). The expression of markers was considered according to the numerical result of the software image analysis algorithms (SP263) [25,26] (Supplementary Materials). In this line, based on the numerical result of the parameter in pixel, a median between the color intensity and the number of cells with positive staining was obtained [27,28,29] (Supplementary Materials). A hotspot was subjectively defined as the area with the highest density of Ki-67-positive tumor cells. To classify, we categorized Ki-67 into distinct low- and high-proliferating tumors, including up to 15% and greater than 15%, respectively [30] (Supplementary Materials).

### 2.4. Follow-Up

Overall survival (OS) was calculated as the length of time from the date of diagnosis or the start of treatment for a disease (a total of 691 patients), such as cancer, that patients diagnosed with the disease are still alive—a total of 434 alive and 257 deaths [31]. Recurrence-free survival (RFS) was calculated as the measure of time from random assignment to cancer recurrence or death from any cause [32] (Supplementary Materials).

### 2.5. Statistical Analysis

Statistical association between categorical variables was assessed using either the Chi-squared test or Fisher’s exact test. Locoregional relapse was defined as the occurrence of any local or regional recurrence and/or death resulting from the tumor. Cox regression analysis was used to perform univariate analyses for overall survival and recurrence-free survival. Hazard ratios with 95% CI were presented. The statistical analysis was conducted using SPSS software version 26 for Windows (SPSS for Windows, 12 Inc., Chicago, IL, USA).

Kaplan–Meier curves for overall survival and recurrence-free survival were generated within the R statistical environment version 4.3.0 using the univariate log rank (Mantel–Cox) test. A *p*-value threshold of 0.05 was considered statistically significant.

## 3. Results

### 3.1. Clinicopathologic Features of Patients

The characteristics of 691 patients are summarized in Table 1 and Table S2. The mean age was 52.5 years with 40.2% aged between 40 and 60. FIGO and TNM had more patients in stage I, 56.9% and 53.5%, respectively; 28.1% relapsed, and 37.2% died within 5 years (Table 1). Of those 468 patients indicated for surgical treatment, 41.10% had a tumor size larger than 4 cm, 43.4% presented angiolymphatic invasion, 10.5% with neural invasion, and 51.7% showed deeper stromal invasion. Lymph node metastasis was found in 26.7% and a positive resection margin in 9.6%. The most frequent pathological stage found was stage I in 58.8% (Table S3).

### 3.2. Analysis of Survival Rate, Clinicopathological Features, and Age

Univariate logistic Cox regression analysis was used to investigate the impact of age, TNM, and FIGO staging on death rates. Patients had hazard ratios of 3.67 (95% CI 1.98 to 6.80), 8.89 (95% CI 5.31 to 14.88), and 10.95 (95% CI 5.31 to 14.88) for older age, FIGO stage IV, and TNM stage, respectively. A significant statistical correlation was found between survival and age (years, <40: *p* < 0.0001; >40 and <60: *p* < 0.05; >60 and <80: *p* < 0.0001 and >80: *p* < 0.0001), FIGO staging (all categories, *p* < 0.0001), and TNM staging (all categories, *p* < 0.0001) (Table 2).

Furthermore, survival had significant statistical correlation with hemoglobin level (g/dL, until >8–12: *p* < 0.0001;), leukocytes count (leukocytosis: *p* < 0.0001), thrombocytosis (*p* < 0.0001), tumor size (>4 cm: *p* < 0.0001), positive HPV (*p* < 0.0001), neural and angiolymphatic invasion (*p* < 0.05); depth of stromal invasion (*p* < 0.0001), lymph node metastasis (*p* < 0.0001), number of resected iliac pelvic and retroperitoneal lymph nodes (6–10: *p* < 0.05 and >3: *p* < 0.05, respectively), TNM pathological Staging (IV: *p* < 0.0001), and chemotherapy (*p* < 0.01) (Table S4).

### 3.3. A Strong Association Interaction between CD204+ and CD163+ M2-TAM with STAT3 and NF-κB Signaling Pathways

The association correlation between the expression of CD204+ and CD163+ M2-TAM with STAT3/NF-κB signaling pathways in the tumor microenvironment (TME) was studied in 691 patients with cervical cancer (Table 3). Importantly, the correlation was strongly significant to STAT3 and NF-κB transcription factors (all *p* < 0.0001), showing that the higher expression of STAT3 was related to higher expression of NF-κB, CD163, and CD204 (Table 3). Cytoplasmic staining of NF-κB transcription factor and CD163 were identified in 65.8% and 65.6% of 691 patients with cervical cancer, respectively (Table 3 and Figure 2). On the other hand, STAT3 transcription factor and CD204 showed cytoplasmic/nuclear staining present in 85.4% and 85.6% of cases (Table 3 and Figure 2).

### 3.4. Transcription Factors (STAT3 and NF-κB) and M2-TAM (CD204 and CD163) Modulated EMT (Vimentin, E-Cadherin, and SNAIL) and Invasion (MMP9)

Protein expression of Vimentin (VIM), E-Cadherin (E-cad), MMP9, and SNAIL was correlated with STAT3, NF-κB, CD163, and CD204. Higher expression of E-Cadherin, MMP9, Vimentin (all *p* < 0.0001), and SNAIL (*p* < 0.001) significantly correlated with higher expression of STAT3 (Table 4). Higher expression of MMP9 (*p* < 0.0001), Vimentin (*p* < 0.0001), and E-cadherin (*p* < 0.05), but not SNAIL (*p* < 0.05), significantly correlated with higher expression of NF-κB (Table 4). Higher expression of CD163 was crucial to the high expression of MMP9 (*p* < 0.0001), Vimentin (*p* < 0.0001), E-cadherin (*p* < 0.0001), but not SNAIL (*p* > 0.05). Higher expression of STAT3, NF-κB, CD204, and CD163 had a strong correlation with higher expression of CD204 (all *p* < 0.0001 and SNAIL *p* < 0.05) (Table 5).

**Figure 3:**
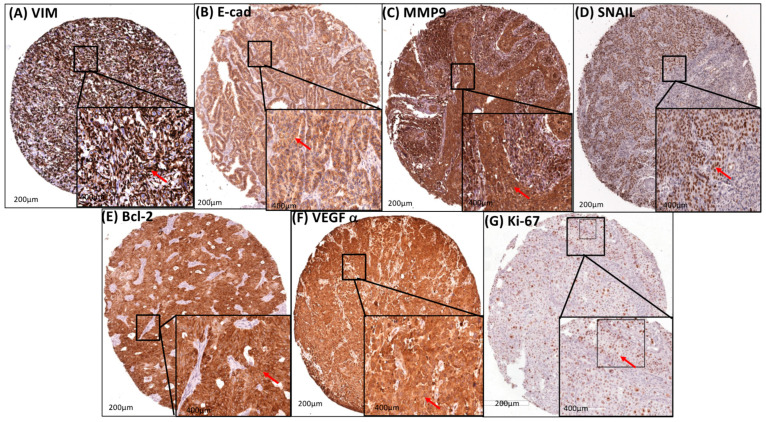
Protein expression profiles of cervical tumors to evaluate EMT, Invasion, Apoptosis, Angiogenesis, and Cell Proliferation. (**A**) diffuse brownish-brown coloration with strong cytoplasmic/membrane staining for anti-VIM (red narrow), (**B**) diffuse brownish-brown coloration with strong cytoplasmic/membrane for anti-E-cad (red narrow), (**C**) diffuse brownish-brown coloration with strong cytoplasmic staining/nuclear staining for anti-MMP9 (red narrow), (**D**) diffusely brownish-brown staining with strong nuclear staining for anti-SNAIL (red narrow), (**E**) diffusely brownish-brown staining with strong cytoplasmic/nuclear staining for anti-Bcl-2 (red narrow), (**F**) diffusely brownish-brown staining with strong cytoplasmic staining for anti-VEGFα (red narrow), and (**G**) restricted brownish-brown staining with some strong nuclear staining for anti-Ki-67 (red narrow) on Tissue Microarray (TMA) with primary cervical cancer. The immunohistological profile presents images at 200× magnification; 400× magnification is shown in the lower right corner of each image.

The cytoplasmic/membrane staining of Vimentin and E-cadherin was seen in 84.1% and 65.6% of the cases, respectively. The cytoplasmic/nuclear staining of MMP9 was observed in 89.4%, and the nuclear staining of SNAIL was found in 55% of 691 patients (Table 4 and Table 5, and Figure 3).

### 3.5. Transcription Factors (STAT3 and NF-κB) and M2-TAM (CD204 and CD163) Upregulated the Immunosuppression in the Cervical Cancer TME

**Figure 4:**
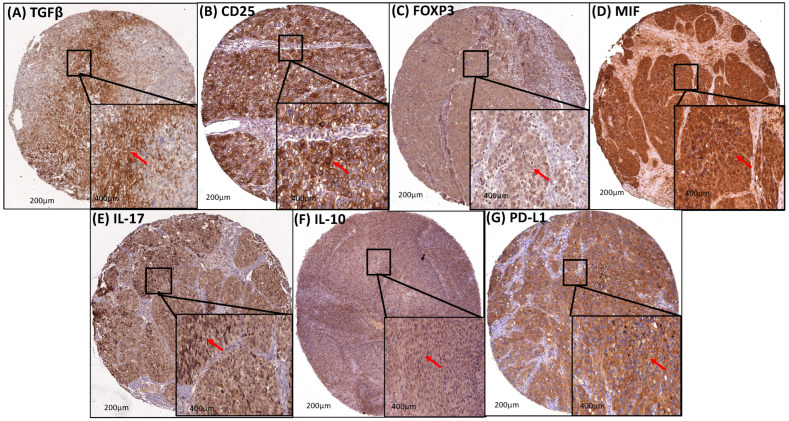
Protein expression profiles of cervical tumors to assess immunosuppression. Strong diffuse brownish-brown cytoplasmic staining for anti-TGFβ (**A**), anti-CD25 (**B**), anti-FOXP3 (**C**), anti-MIF (**D**), anti-IL-17 (**E**), anti-IL-10 (**F**), and anti-PD-L1 (**G**) in TMA with primary cervical cancer. Red arrow: brownish-brown cytoplasmic staining.

The staining of TGFβ, PD-L1, IL-10, FOXP3, IL-17, and MIF was cytoplasmic and present in 25%, 9.4%, 56.2%, 7.2%, 76.3%, 86.1%, and 85.2%, respectively, of the cervical cancer cases (Table 6 and Table 7, and Figure 4). Furthermore, these immunosuppressive markers were correlated with the expression of STAT3, NF-κB, CD163, and CD204. On the other hand, it was statistically significant with the higher expression of CD163 (*p* < 0.05) and CD204 (*p* < 0.05). Higher expression of CD25, FOXP3, IL-17, and MIF was strongly associated with the higher expression of STAT3, NF-κB, CD163, and CD204 (all *p* < 0.0001). However, the higher expression of IL-10 was associated with the expression of STAT3 (*p* < 0.001), CD163 (*p* < 0.05), and CD204 (*p* < 0.05) but not with NF-κB. Similarly, the association between the higher expression of PD-L1 was statistically significant with the higher expression of STAT3 (*p* < 0.001) and CD204 (*p* < 0.007) but not with NF-κB and CD163 (Table 6 and Table 7, and Figure 4).

### 3.6. Transcription Factors (STAT3 and NF-κB) and M2-TAM (CD204 and CD163) Had a Strong Association Correlation with Apoptosis, Angiogenesis, and Proliferation

The cytoplasmic/nuclear staining of Bcl-2 and the cytoplasmic staining of VEGFα were present in 77.4% and 78.4%, respectively (Table 8 and Table 9, and Figure 3). On the other hand, the nuclear staining of Ki-67 was found in 66.3% (Table 8 and Table 9, and Figure 3). There was a strong association between the higher expression of VEGFα and the expression of STAT3, NF-κB, CD163, and CD204 (all *p* < 0.0001). Likewise, the higher expression of Bcl-2 was statistically significant with higher expression of STAT3, NF-κB, CD163, and CD204 (all *p* < 0.0001 and NF-κB *p* < 0.05). And finally, the expression of Ki-67 was only statistically significant with NF-κB expression (*p* < 0.001) (Table 8 and Table 9).

### 3.7. Assessment of Overall Survival and Recurrence-Free Survival of Patients with Cervical Cancer Based on Protein Expression of STAT3, NF-κB, CD163, and CD204

**Figure 5:**
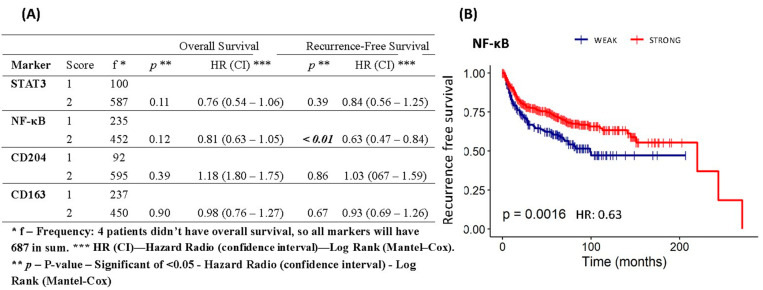
Survival analysis of the risk model by expression of STAT3, NF-κB, CD204, and CD163 proteins in 691 patients with cervical cancer. (**A**) Comparison of the predictive power of the multiple risk model using the results of Cox regression in overall survival and recurrence-free survival, significance is seen only for NF-κB. (**B**) Kaplan–Meier recurrence-free survival curve estimated by NF-κB staining of patients with cervical cancer after treatment with worse prognosis for weak staining (log-rank χ^2^ = 0.63; *p* = 0.0016).

Based on Kaplan–Meier analysis, the STAT3, CD163, and CD204 expression had no significant correlation with the overall survival rate and recurrence-free rate, except the NF-κB expression (*p* < 0.01) that had a significant correlation with a recurrence-free survival rate. The patients who showed a higher NF-κB expression had a significantly more favorable outcome in recurrence-free survival than those with a lower expression (Figure 5).

### 3.8. Assessment of Overall Survival and Recurrence-Free Survival Evaluations of Patients with Cervical Cancer Based on Protein Expression of EMT, Invasion, Immunosuppression, Resistance to Apoptosis, Angiogenesis, and Proliferation

**Figure 6:**
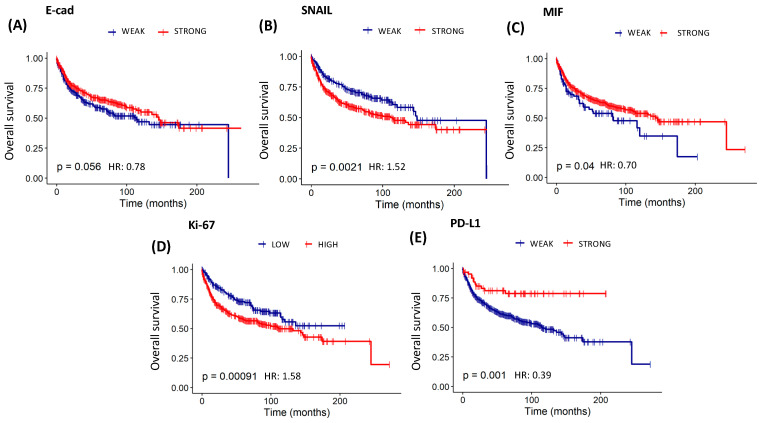
Survival analysis of the risk model by E-cad, SNAIL, MIF, Ki-67, and PD-L1 protein expression in 691 patients with cervical cancer. Kaplan–Meier Overall Survival Curve estimated by staining of (**A**) E-cad (log-rank χ^2^ = 0.78; *p* = 0.056), (**B**) SNAIL (log-rank χ^2^ = 1.52; *p* = 0.0021), (**C**) MIF (log-rank χ^2^ = 0.70; *p* = 0.04), (**D**) Ki-67 (log-rank χ^2^ = 1.58; *p* = 0.00091), and (**E**) PD-L1 (log-rank χ^2^ = 0.39; *p* = 0.001).

**Figure 7:**
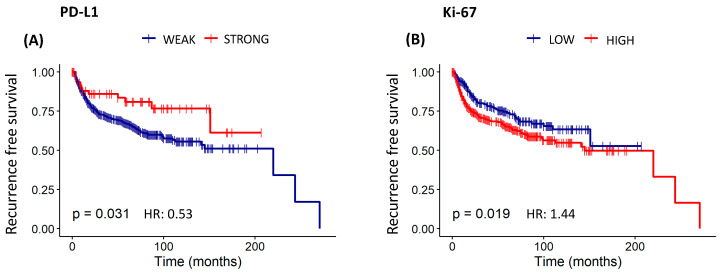
Survival analysis of the risk model by PD-L1 and Ki-67 protein expression in 691 patients with cervical cancer. Kaplan–Meier Recurrence-Free Survival Curve estimated by staining of (**A**) PD-L1 (log-rank χ^2^ = 0.53; *p* = 0.031), and (**B**) Ki-67 (log-rank χ^2^ = 1.44; *p* = 0.019).

The study found statistically significant overall survival curves for E-cad (*p* < 0.05), SNAIL (*p* < 0.01), PD-L1 (*p* < 0.01), MIF (*p* < 0.05), and Ki-67 (*p* < 0.001) (Table 10 and Figure 6). Patients with a higher expression of E-cad (HR: 0.78) and MIF (HR: 0.70), as well as a lower expression of PD-L1 (HR: 0.39), had a significantly more favorable outcome when overall survival was analyzed. On the other hand, patients who showed a higher expression of SNAIL (HR: 1.52) and Ki-67 (HR: 1.58) had a significantly less favorable outcome in overall survival. Likewise, statistically significant recurrence-free survival curves to PD-L1 and Ki-67 (both *p* < 0.05) were found, pointing out that patients with a lower expression of PD-L1 (HR: 0.53) had a higher disease-free chance. On the other hand, those who had a higher expression of Ki-67 (HR: 1.44) showed a higher recurrence risk (Table 10 and Figure 7).

### 3.9. Association Correlation between Transcription Factors, EMT, Invasion, Immunosuppression, Apoptosis Resistance, Angiogenesis, and Proliferation with Clinical TNM Staging

Based on Fisher’s exact test analysis, the protein expression of STAT3 and NF-κB (both *p* < 0.01) had a significant correlation with TNM staging (Table 11). Furthermore, protein expression of VIM, E-cad, SNAIL, CD25, MIF, PD-L1, VEGFα, and Ki-67 had statistically high significance with TNM staging (*p* < 0.001, *p* < 0.0001, *p* < 0.0001, *p* < 0.001, *p* < 0.0001, *p* < 0.001, *p* < 0.001, and *p* < 0.0001, respectively) (Table S5).

### 3.10. Prognostic Significance of Patients by TNM Clinic Staging

**Figure 8:**
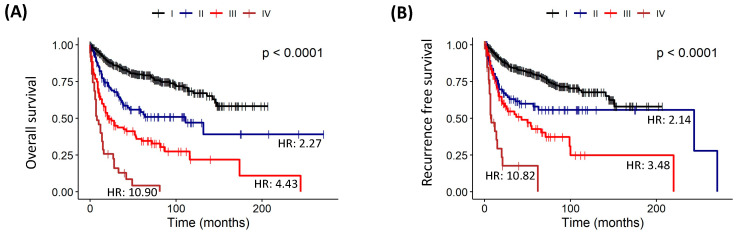
Survival analysis of the risk model by Clinical TNM Staging in 691 patients with cervical cancer. Kaplan–Meier Overall Survival Curve estimated by TNM (I-IV) (**A**) and Kaplan–Meier Recurrence-Free Survival Curve estimated by TNM (I-IV) (**B**).

In this study, the relationship between the TNM staging and death had a statistically significant correlation when overall survival and recurrence-free survival curves were studied using the Kaplan–Meier analysis (all *p* < 0.0001). In this context, patients with a higher stage (III and IV) had a significantly less favorable outcome in overall survival (III-HR: 4.43; IV-HR: 10.90) and recurrence-free survival (III-HR: 3.48; IV-HR: 10.82) (Table 12, and Figure 8).

### 3.11. Overall Survival of Patients with Cervical Cancer Based on Lifestyle Database, Laboratory Analysis, Treatment, and Clinicopathological Characteristics

Additionally, this study assessed the impact of clinicopathological variables on the overall survival of patients with cervical cancer. Table 13 summarizes the variables that increased the death risk. Interestingly, leukocytosis, thrombocytosis, therapy, neural invasion, compromised margin, and lymph node metastasis were independent prognostic factors for OS, enhancing the hazard of death (HR: 1.36–2.98). On the other hand, deeper stromal invasion, tumor size higher than 4 cm, and TNM pathological stage showed to be important factors to increase the hazard of death at 3.43, 4.32, and 7.58, respectively (Table 13 and Table S4).

## 4. Discussion

In this study, we found that a high degree of M2-TAM infiltration and the activation of STAT3/NF-κB signaling in TME of cervical cancer was associated with the upregulation of expression of protein factors related to immunosuppression, EMT, invasion, resistance to apoptosis, angiogenesis, and proliferation, which resonated in adverse clinical and pathological prognostic parameters. The interaction of M2-TAM and via STAT3/NF-κB with the tumor cells in TME creates a hostile environment for the immune cells but a favorable one for tumor progression [19,21]. In this context, we hypothesized that M2-TAMs and via STAT3/NF-κB crosstalk can modulate important hallmarks of tumor progression, negatively affecting the survival of 691 patients with cervical cancer.

The development of tumor progression by M2-TAM involves a complex interaction of signals, including stroma cells through cytokines, and chemokines, which stimulates the crosstalk of the STAT-3 pathway with NF-κB [33,34]. In TME, the crosstalk of STAT3 and NF-κB pathways promotes an important role in modulating cancer-associated inflammation-promoting TAM polarization towards M2, as well as stimulating resistance to apoptosis, EMT, invasion, and proliferation in tumor cells [35,36,37]. As clinical relevance, the increased expression of STAT3 and NF-κB has been associated with higher clinical TNM staging in solid tumors, which was confirmed in our study [38,39]. Furthermore, we found a strong association correlation between +CD163 + CD204M2-TAM with STAT3 and NF-κB signaling pathways, as well as with factors linked to tumor growth, dissemination, and immunosuppression [40,41,42,43,44].

EMT type III, EMT associated with metastasis, is an important event in the evolution of cancer that is induced by a set of signaling molecules, such as STAT3 and NF-κB, and signaling cell (M2-TAM) [20,45]. Collectively, they stimulate multiple signaling pathways, thereby activating a small set of transcription factors (TFs), for example, SNAIL, or master regulators of EMT. These TFs act to suppress the expression of epithelial markers such as E-cadherin, as well as activation of mesenchymal markers such as N-cadherin, Vimentin, matrix metalloproteinases, favoring tumor infiltration, dissemination, and metastasis associated with increased resistance to apoptosis (bcl-2) contributing to the poor prognosis of patients [45,46].

Here, the expression of E-cadherin, Vimentin, and SNAIL had a relevant correlation with the overall survival of 691 patients with cervical cancer. The loss of the cell–cell junction by lower expression of E-cadherin changes the cytoskeleton of epithelial cells to a mesenchymal phenotype, leading to higher expression of Vimentin and SNAIL, a transcription factor of EMT process [47,48,49]. These EMT biomarkers are remarkable factors of worse prognosis [50,51,52]. Furthermore, M2-TAM regulates the deposition or degradation of ECM components by secreting matrix metallopeptidase (MMPs), serine, and cathepsins, which contributes to the tumor dissemination [53,54,55,56]. Our results showed that CD163 + CD204 + M2-TAM via STAT3/NF-κB signaling pathways was strongly responsible for increasing protein factors of EMT and invasion (MMP9), reverberating in a worse prognosis scenario in patients with cervical cancer.

Resistance to apoptosis, proliferation, and angiogenesis are cancer hallmarks that contribute to the tumor growth and dissemination predicting evolution of clinical outcomes [57,58,59]. There is strong evidence that the interaction between cancer-specific Bcl-2, STAT3/NF-κB signaling and the recruitment of M2-TAM promote a pro-tumor microenvironment [21,60]. Likewise, M2-TAMs produce pro-angiogenic factors, such as VEGFα, to improve angiogenesis in TME and promote the tumor expansion [61]. In addition, the nuclear Ki-67 protein is generally expressed only in proliferating cells that are from G1 phase to mitosis, and its expression rapidly decreases immediately after mitosis [62]. For that, the immunostaining for Ki-67 expression is the gold standard, and a cutoff level of 10–14% positive staining is used to judge high risk of prognosis [63]. Our results showed a strong association correlation of CD163 + CD204 + M2-TAM and via STAT3/NF-κB signaling pathways with the higher expression of Bcl-2 and VEGFα, as well as with elevated index of Ki-67 (>15% positive staining). And more interestingly, the higher Ki-67 proliferation index found in our study was associated to higher clinical TNM stage (III and IV), disease recurrence, and hazard of death in patients with cervical cancer, which indicated a strong corroboration with reports in the literature [64,65,66].

In metastasis, NF-κB cooperates with STAT3 in promoting cancer progression, conferring resistance to apoptosis and plasticity. Furthermore, NF-κB and STAT3 interact and cooperate in regulating the interaction of malignant cells and the tumor microenvironment, especially immune cells such as TAMs that release cell-stimulating growth factors and cytokines, including IL-6, IL-10, and TNF-α [67,68,69].

In the TME, M2-TAMs exert an immunosuppressive function that acts on the positive regulation of the STAT3 pathway and the negative regulation of NF-κB in cells of the immune system [19,67,70,71,72]. In this line, macrophage migration inhibitory factor (MIF) has been implicated to upregulate STAT3 pathway in monocyte/macrophage towards M2-TAM [73]. Regulatory T cells (Treg cells) have been recognized as a core component of immunosuppression by suppressing cytotoxic T cells in many tumors [74]. FOXP3, a member of the Forkhead/winged-helix family of transcriptional regulators, converts naive T cells (CD4 + CD25-T cells) into suppressive inducible Foxp3+ Treg (CD4 + CD25 + FOXP3 + T cells) when regulated by TGF-β, IL-17, IL-10, and PD-L1 [75,76,77]. A high expression of CD25, a surface receptor of Treg cells, and FOXP3, as well as a low level of CD8^+^ T cells in the TME are associated with unfavorable prognosis [16,19,78]. Interestingly, our results showed a strong association correlation between CD163 + CD204 + M2TAM, via STAT3/NF-κB pathways and the expression of CD25, FOXP3, MIF, and IL-17 in 56.2%, 76.3%, 85.2%, and 86.1%, respectively, of 691 patients with cervical cancer. This expression profile, especially for CD25 and MIF, reverberated in a higher clinical TNM staging (III and IV) and consequently in an increased hazard of death and disease recurrence. On the other hand, the weak expression of TGFβ, IL-10, and PD-L1 in 75%, 92.8%, and 90.6% of patients did not have any association correlation with CD163 + 204 + M2-TAM, via STAT3 and NF-κB pathways or with clinical–pathological parameters. These weak labeling results may limit our analyses into the immunosuppressive role of these signaling pathways for the outcome of death. There are also interactions with alternative pathways that will act on tumor progression. Some reports describe that the tumor immune escape can be driven by other immunosuppressive cytokine loops, including CCL22, CCL17, and CCL18 that are also secreted by macrophage for recruiting Treg cells and modulate the immune response during the tumorigenic process [79,80].

An immunosuppressive molecular scenario reverberates often in an unfavorable clinical–pathological profile, on treatment failure and death. Some studies point out that older women, particularly those over age 70 years, independent of other factors such as comorbidities, histology, and stage of disease, show significantly decreased survival indexes [81,82,83]. In our study, patients of advanced age, 60–70 years and >80 years, presented a higher hazard of death (2.33 and 3.67, respectively). Pathological type, tumor size, FIGO stage, and lymph node metastasis are parameters related to recurrence and overall survival [84]. However, the criteria of lymph node metastasis seen in pathological TNM staging is considered an independent prognostic factor for overall survival in advanced disease, being used to guide postoperative adjuvant therapy [85]. In this line, our results showed that tumor size > 4 cm, FIGO staging IV, lymph node metastasis, and TNM pathological staging IV showed a strong correlation with overall survival (HR: 2.81–8.89). Furthermore, other parameters investigated, such as leukocytosis, thrombocytosis, depth of stromal invasion, and compromised margin, were relevant factors for death with HR ranging from 2.46 to 3.43 [86,87]. Paradoxically, overactivity of the immune system may allow the tumor evasion from the immune response by the tumor using specific immune cells known for their immunosuppressive activity [88]. In this context and as described previously, an elevated expression of Treg cells in TME and leukocytosis were observed in patients with locally advanced cervical cancer seen in cases of the higher clinical–pathological TNM staging (III and IV), reverberating in an elevated risk of death. Therefore, our study strongly reported that tumor progression is associated with the high expression of M2-TAM and STAT3/NF-κB pathways, leading to lower survival in cervical cancer. On the other hand, a weak association of analyzed immunosuppressive cytokines with the tumor progression and survival was a limitation of this study.

## 5. Conclusions

M2-TAM and STAT3/NF-κB pathways upregulated the expression of immunosuppressive components, leading to a stimulatory loop in EMT, invasion, angiogenesis, apoptosis resistance, and proliferation, which negatively reverberated on the clinic parameters, including TNM stage, disease recurrence, and death in 691 patients with cervical cancer. These findings showed that protein biomarkers related to the interaction of M2-TAM and cancer cells in TME have a strong predictive value in cervical cancer. Therefore, these results can act as new protein biomarkers that will contribute to new lines of oncological treatment.

## Figures and Tables

**Table 1:** Distribution of 691 patients with cervical cancer by age, clinical stage, recurrence, and survival.

Clinicopathological Features	Category	Frequency	Percentage
Age (Years)	≤40	184	26.6%
	>40 and ≤60	278	40.2%
	>60 and ≤80	201	29.1%
	>80	27	3.9%
	Not available	1	0.1%
	Total Number	691	100.0%
FIGO Staging	I	393	56.9%
	II	122	17.7%
	III	101	14.6%
	IV	21	3.0%
	Not available	54	7.8%
	Total Number	691	100.0%
TNM Staging	I	370	53.5%
	II	109	15.8%
	III	106	15.3%
	IV	37	5.4%
	Not available	69	10.0%
	Total Number	691	100.0%
Recurrence	No	460	66.6%
	Yes	194	28.1%
	Not available	37	5.4%
	Total Number	691	100.0%
Death	No	417	60.3%
	Yes	257	37.2%
	Not available	17	2.5%
	Total Number	691	100.0%

**Table 2:** Cox regression for overall survival to clinical staging.

Clinicopathological Features	Category	Freq *	Significance **	Hazard Ratio of Exp(*B*) ***
Age (Years)	<=40	182	<0.0001	
	>40 and <=60	277	<0.05	1.50 (1.06–2.12)
	>60 and <=80	200	<0.0001	2.33 (1.63–3.31)
	>80	27	<0.0001	3.67 (1.98–6.80)
FIGO Staging	I	391	<0.0001	
System	II	122	<0.0001	2.44 (1.74–3.41)
	III	100	<0.0001	5.68 (4.15–7.79)
	IV	20	<0.0001	8.89 (5.31–14.88)
TNM Staging System	I	369	<0.0001	
	II	109	<0.0001	2.29 (1.60–3.28)
	III	105	<0.0001	4.47 (3.23–6.17)
	IV	36	<0.0001	10.95 (7.22–16.62)

* Frequency; ** Significant (*p* < 0.05); *** Log Rank (Mantel–Cox).

**Table 3:** Protein expression of STAT3, NF-κB, CD163, and CD204 in primary cervical tumors and the association correlation between them.

Marker			STAT3			NF-κB		
	Immunoexpression		1	2		1	2	
			Weak *n* [%] = 101 [14.6]	Strong *n* [%] = 590 [85.4]	*p* *	Weak *n* [%] = 236 [34.2]	Strong *n* [%] = 455 [65.8]	*p* *
NF-κB	1	Weak n [%] = 236 [34.2]	62 [9.0]	174 [25.2]	<0.0001	-		-
	2	Strong n [%] = 455 [65.8]	39 [5.6]	416 [60.2]				
CD163	1	Weak n [%] = 238 [34.4]	68 [9.8]	170 [24.6]	<0.0001	118 [17.1]	120 [17.4]	<0.0001
	2	Strong n [%] = 453 [65.6]	33 [4.8]	420 [60.8]		118 [17.1]	335 [48.5]	
CD204	1	Weak n [%] = 93 [13.5]	51 [7.4]	42 [6.1]	<0.0001	48 [6.9]	45 [6.5]	<0.0001
	2	Strong n [%] = 598 [86.5]	50 [7.2]	548 [79.3]		188 [27.2]	410 [59.3]	

Fisher’s exact test (* Significant *p* < 0.05); Abbreviations: STAT3 = Signal Transducer and Activator of Transcription 3; NF-κB = Nuclear Factor-kappa B and M2-TAM = Tumor-associated Macrophages Type 2.

**Table 4:** Protein expression of vimentin, e-cadherin, matrix metalloproteinase 9, SNAIL, STAT3, and NF-κB in primary cervical tumors and the association correlation between them.

Marker			STAT3			NF-κB		
	Immunoexpression		1	2		1	2	
			Weak *n* [%] = 101 [14.6]	Strong n [%] = 590 [85.4]	*p* *	Weak *n* [%] = 236 [34.2]	Strong *n* [%] = 455 [65.8]	*p* *
VIM	1	Weak n [%] = 110 [15.9]	58 [8.4]	52 [7.5]	<0.0001	68 [9.8]	42 [6.1]	<0.0001
	2	Strong n [%] = 581 [84.1]	43 [6.2]	538 [77.9]		168 [24.3]	413 [59.8]	
E-cad	1	Weak n [%] = 311 [45.0]	79 [11.4]	232 [33.6]	<0.0001	122 [17.7]	189 [27.4]	<0.05
	2	Strong n [%] = 380 [55.0]	22 [3.2]	358 [51.8]		114 [16.5]	266 [38.5]	
MMP9	1	Weak n [%] = 73 [10.6]	47 [6.8]	26 [3.8]	<0.0001	44 [6.4]	29 [4.2]	<0.0001
	2	Strong n [%] = 618 [89.4]	54 [7.8]	564 [81.6]		192 [27.8]	426 [61.6]	
SNAIL	1	Weak n [%] = 238 [34.4]	47 [6.8]	191 [27.6]	<0.01	78 [11.3]	160 [23.2]	0.61
	2	Strong n [%] = 453 [65.6]	54 [7.8]	399 [57.7]		158 [22.9]	295 [42.7]	

* Fisher’s exact test (Significant *p* < 0.05); Abbreviations: STAT3 = Signal Transducer and Activator of Transcription 3; NF-κB = Nuclear Factor-kappa B; VIM = Vimentin; E-cad = E-cadherin; and MMP9 = Matrix Metalloproteinase-9.

**Table 5:** Protein expression of vimentin, E-cadherin, matrix metalloproteinase 9, and SNAIL in primary cervical tumors and the association correlation with M2-TAM (CD163 and CD204).

Marker			CD163			CD204		
	Immunoexpression		1	2		1	2	
			Weak *n* [%] = 238 [34.4]	Strong *n* [%] = 453 [65.6]	*p* *	Weak *n* [%] = 93 [13.5]	Strong *n* [%] = 598 [86.5]	*p* *
VIM	1	Weak n [%] = 110 [15.9]	77 [11.1]	33 [4.8]	<0.0001	56 [8.1]	54 [7.8]	<0.0001
	2	Strong n [%] = 581 [84.1]	161 [23.3]	420 [60.8]		37 [5.4]	544 [78.7]	
E-cad	1	Weak n [%] = 311 [45.0]	131 [19.0]	180 [26.0]	<0.0001	68 [9.8]	243 [35.2]	<0.0001
	2	Strong n [%] = 380 [55.0]	107 [15.5]	273 [39.5]		25 [3.6]	355 [51.4]	
MMP9	1	Weak n [%] = 73 [10.6]	53 [7.7]	20 [2.9]	<0.0001	44 [6.4]	29 [4.2]	<0.0001
	2	Strong n [%] = 618 [89.4]	185 [26.8]	433 [62.7]		49 [7.1]	569 [82.3]	
SNAIL	1	Weak n [%] = 238 [34.4]	92 [13.3]	146 [21.1]	0.09	41 [5.9]	197 [28.5]	<0.05
	2	Strong n [%] = 453 [65.6]	146 [21.1]	307 [44.4]		52 [7.5]	401 [8.0]	

* Fisher’s exact test (Significant *p* < 0.05); Abbreviations: VIM = Vimentin; E-cad = E-cadherin; MMP9 = Matrix Metalloproteinase 9; and M2-TAM = Tumor-associated Macrophages Type 2.

**Table 6:** Protein expression of TGFβ, CD25, FOXP3, MIF, IL-17, IL-10, and PD-L1 in primary cervical tumors and the association correlation with STAT3 and NF-κB.

Marker			STAT3			NF-κB		
	Immunoexpression		1	2		1	2	
			Weak *n* [%] = 101 [14.6]	Strong *n* [%] = 590 [85.4]	*p* *	Weak *n* [%] = 236 [34.2]	Strong *n* [%] = 455 [65.8]	*p* *
TGFβ	1	Weak n [%] = 518 [75.0]	80 [11.6]	438 [63.4]	0.25	180 [26.0]	338 [48.9]	0.58
	2	Strong n [%] = 173 [25.0]	21 [3.0]	152 [22.0]		56 [8.1]	117 [16.9]	
CD25	1	Weak n [%] = 303 [43.8]	74 [10.7]	229 [33.1]	<0.0001	137 [19.8]	166 [24.0]	<0.0001
	2	Strong n [%] = 388 [56.2]	27 [3.9]	361 [52.2]		99 [14.3]	289 [41.8]	
FOXP3	1	Weak n [%] = 164 [23.7]	66 [9.6]	98 [14.2]	<0.0001	72 [10.4]	92 [13.3]	<0.0001
	2	Strong n [%] = 527 [76.3]	35 [5.1]	492 [71.2]		164 [23.7]	363 [52.5]	
MIF	1	Weak n [%] = 102 [14.8]	60 [8.7]	42 [6.1]	<0.0001	51 [7.4]	51 [7.4]	<0.0001
	2	Strong n [%] = 589 [85.2]	41 [5.9]	548 [79.3]		185 [26.8]	404 [58.5]	
IL-17	1	Weak n [%] = 96 [13.9]	56 [8.1]	40 [5.8]	<0.0001	57 [8.2]	39 [5.6]	<0.0001
	2	Strong n [%] = 595 [86.1]	45 [6.5]	550 [79.6]		179 [25.9]	416 [60.2]	
IL-10	1	Weak n [%] = 641 [92.8]	101 [14.6]	540 [78.1]	<0.001	220 [31.8]	421 [60.9]	0.87
	2	Strong n [%] = 50 [7.2]	0 [0.0]	50 [7.2]		16 [2.3]	34 [4.9]	
PD-L1	1	Weak n [%] = 626 [90.6]	100 [14.5]	526 [76.1]	<0.001	210 [30.4]	416 [60.2]	0.33
	2	Strong n [%] = 65 [9.4]	1 [0.1]	64 [9.3]		26 [3.8]	39 [5.6]	

* Fisher’s exact test (Significant *p* < 0.05); Abbreviations: TGFβ = Transforming growth factor β; CD25 = α-chain of IL-2 receptor; IL-10 = Interleukin 10; IL-17 = Interleukin 17; PD-L1 = Programmed cell death-ligand 1; FOXP3 = Forkhead box protein P3; MIF = macrophage migration inhibitory factor.

**Table 7:** Protein expression of TGFβ, CD25, FOXP3, MIF, IL-17, IL-10, and PD-L1 in primary cervical tumors and the association correlation with M2-TAM (CD163 and CD204).

Marker			CD163			CD204		
	Immunoexpression		1	2		1	2	
			Weak *n* [%] = 238 [34.4]	Strong *n* [%] = 453 [65.6]	*p* *	Weak *n* [%] = 93 [13.5]	Strong *n* [%] = 598 [86.5]	*p* *
TGFβ	1	Weak n [%] = 518 [75.0]	191 [27.6]	327 [47.3]	<0.05	80 [11.6]	438 [63.4]	<0.01
	2	Strong n [%] = 173 [25.0]	47 [6.8]	126 [18.2]		13 [1.9]	160 [23.2]	
CD25	1	Weak n [%] = 303 [43.8]	137 [19.8]	166 [24.0]	<0.0001	69 [10.0]	234 [33.9]	<0.0001
	2	Strong n [%] = 388 [56.2]	101 [14.6]	287 [41.5]		24 [3.5]	364 [52.7]	
FOXP3	1	Weak n [%] = 164 [23.7]	85 [12.3]	79 [11.4]	<0.0001	64 [9.3]	100 [14.5]	<0.0001
	2	Strong n [%] = 527 [76.3]	153 [22.1]	374 [54.1]		29 [4.2]	498 [72.1]	
MIF	1	Weak n [%] = 102 [14.8]	59 [8.5]	43 [6.2]	<0.0001	44 [6.4]	58 [8.4]	<0.0001
	2	Strong n [%] = 589 [85.2]	179 [25.9]	410 [59.3]		49 [7.1]	540 [78.1]	
IL-17	1	Weak n [%] = 96 [13.9]	69 [10.0]	27 [3.9]	<0.0001	55 [8.0]	41 [5.9]	<0.0001
	2	Strong n [%] = 595 [86.1]	169 [24.5]	426 [61.6]		38 [5.5]	557 [80.6]	
IL-10	1	Weak n [%] = 641 [92.8]	228 [33.0]	413 [59.8]	<0.05	91 [13.2]	550 [79.6]	<0.05
	2	Strong n [%] = 50 [7.2]	10 [1.4]	40 [5.8]		2 [0.3]	48 [6.9]	
PD-L1	1	Weak n [%] = 626 [90.6]	223 [32.3]	403 [58.3]	0.054	91 [13.2]	535 [77.4]	<0.01
	2	Strong n [%] = 65 [9.4]	15 [2.2]	50 [7.2]		2 [0.3]	63 [9.1]	

* Fisher’s exact test (Significant *p* < 0.05); Abbreviations: TGFβ = Transforming growth factor β; CD25 = α-chain of IL-2 receptor; IL-10 = Interleukin 10; IL-17 = Interleukin 17; PD-L1 = Programmed cell death-ligand 1; FOXP3 = Forkhead box protein P3; MIF = macrophage migration inhibitory.

**Table 8:** Protein expression of Bcl-2, VEGFα, and Ki-67 and the association correlation with STAT3 and NF-κB.

Marker			STAT3			NF-κB		
	Immunoexpression		1	2		1	2	
			Weak *n* [%] = 101 [14.6]	Strong *n* [%] = 590 [85.4]	*p* *	Weak *n* [%] = 236 [34.2]	Strong *n* [%] = 455 [65.8]	*p* *
Bcl-2	1	Weak n [%] = 156 [22.6]	51 [7.4]	105 [15.2]	<0.0001	67 [9.7]	89 [12.9]	<0.05
	2	Strong n [%] = 535 [77.4]	50 [7.2]	485 [70.2]		169 [24.7]	366 [53.0]	
VEGFα	1	Weak n [%] = 149 [21.6]	71 [10.3]	78 [11.3]	<0.0001	80 [11.6]	69 [10.0]	<0.0001
	2	Strong n [%] = 542 [78.4]	30 [4.3]	512 [74.1]		156 [22.6]	386 [55.9]	
Ki-67	1	Low n [%] = 223 [33.7]	39 [5.6]	194 [28.1]	0.25	60 [8.7]	173 [25.0]	<0.01
	2	High n [%] = 458 [66.3]	62 [9.0]	396 [57.3]		176 [25.5]	282 [40.8]	

* Fisher’s exact test (significant *p* < 0.05); Abbreviations: VEGFα = vascular endothelial growth factor alfa; Bcl-2 = B-cell lymphoma 2; Ki-67 = marker of proliferation.

**Table 9:** Protein expression of Bcl2, VEGFα, and Ki-67 and the association correlation with M2-TAM (CD163 and CD204).

Marker	Immunoexpression		CD163			CD204		
			1	2		1	2	
			Weak *n* [%] = 238 [34.4]	Strong *n* [%] = 453 [65.6]	*p* *	Weak *n* [%] = 93 [13.5]	Strong *n* [%] = 598 [86.5]	*p* *
Bcl-2	1	Weak n [%] = 156 [22.6]	77 [11.1]	79 [11.4]	<0.0001	46 [6.7]	110 [15.9]	<0.0001
	2	Strong n [%] = 535 [77.4]	161 [23.3]	374 [54.1]		47 [6.8]	488 [70.6]	
VEGFα	1	Weak n [%] = 149 [21.6]	88 [12.7]	61 [8.8]	<0.0001	62 [9.0]	87 [12.6]	<0.0001
	2	Strong n [%] = 542 [78.4]	150 [21.7]	392 [56.7]		31 [4.5]	511 [74.0]	
Ki-67	1	Low n [%] = 223 [33.7]	85 [12.3]	148 [21.4]	0.44	35 [5.1]	198 [28.4]	0.41
	2	High n [%] = 458 [66.3]	62 [9.0]	396 [57.3]		176 [25.5]	282 [40.8]	

* Fisher’s exact test (significant *p* < 0.05); Abbreviations: VEGFα = vascular endothelial growth factor alfa; Bcl-2 = B-cell lymphoma 2; Ki-67 = marker of proliferation.

**Table 10:** Results of univariable Cox regression model analysis on protein expression of EMT, immunosuppression, resistance to apoptosis, angiogenesis, and proliferation.

Marker	Score	*f* *	Overall Survival		Recurrence-Free Survival	
			*p* **	HR (CI) ***	*p* **	HR (CI) ***
VIM	1	109				
	2	578	0.28	0.83 (0.60–1.16)	0.14	0.75 (0.52–1.09)
E-cad	1	309				
	2	378	<0.05	0.78 (0.61–1.00)	0.72	0.95 (0.71–1.26)
MMP9	1	72				
	2	615	0.45	1.19 (0.75–1.88)	0.35	1.29 (0.74–2.22)
SNAIL	1	237				
	2	450	<0.01	1.52 (1.16–2.00)	0.52	1.10 (1.81–1.48)
TGFß	1	515				
	2	172	0.07	1.27 (0.97–1.67)	0.41	1.14 (0.82–1.56)
CD25	1	302				
	2	385	0.40	0.90 (0.70–1.15)	0.43	0.89 (0.66–1.19)
FOXP3	1	163				
	2	524	0.82	0.96 (0.72–1.29)	0.96	0.99 (0.70–1.39)
MIF	1	101				
	2	586	<0.05	0.70 (0.50–0.98)	0.25	0.79 (0.53–1.18)
IL-17	1	95				
	2	592	0.69	0.92 (0.64–1.34)	0.46	0.85 (0.56–1.30)
IL-10	1	637				
	2	50	0.39	1.21 (0.77–1.89)	0.21	0.65 (0.33–1.28)
PD-L1	1	623				
	2	64	0.001	0.39 (0.22–0.70)	<0.05	0.53 (0.29–0.95)
Bcl-2	1	154				
	2	533	0.06	0.76 (0.58–1.01)	0.36	0.85 (0.56–1.30)
VEGFα	1	148				
	2	539	0.07	0.76 (0.57–1.02)	0.06	0.72 (0.51–1.10)
Ki-67	1	232				
	2	455	<0.001	1.58 (1.20–2.09)	<0.05	1.44 (1.05–1.97)

* *f*—Frequency: 4 patients did not attain overall survival, so all markers will have 687 in sum; ** *p*—*p*-value—Significant of <0.05; *** HR (CI)—Hazard Radio (confidence interval)—Log Rank (Mantel–Cox).

**Table 11:** Protein Expression of Transcription Factors (STAT3 and NF-κB) and Immunosuppression by M2-TAM (CD204 and CD163) in primary cervical tumors, and the association correlation with TNM.

Marker	Score	TNM					
		I	II	III	IV	Total	*p* *
STAT3	1	42 (6.8%)	14 (2.3%)	27 (4.3%)	6 (1.0%)	89 (14.3%)	
	2	328 (52.7%)	95 (15.3%)	79 (12.7%)	31 (5.0%)	533 (85.7%)	<0.01
NF-κB	1	109 (17.5%)	44 (7.1%)	48 (7.7%)	13 (2.1%)	214 (34.4%)	
	2	261 (42.0%)	65 (10.5%)	58 (9.3%)	24 (3.9%)	408 (65.6%)	<0.01
CD204	1	49 (7.9%)	10 (1.6%)	21 (3.4%)	5 (0.8%)	85 (13.7%)	
	2	321 (51.6%)	99 (15.9%)	85 (13.7%)	32 (5.1%)	537 (86.3%)	0.15
CD163	1	128 (20.6%)	33 (5.3%)	39 (6.3%)	12 (1.9%)	212 (34.1%)	
	2	242 (38.9%)	76 (12.2%)	67 (10.8%)	25 (4.0%)	410 (65.9%)	0.77

* Fisher’s exact test (significant *p* < 0.05); TNM data are missing in 96 patients. In this analysis, 622 of them were categorized.

**Table 12:** Cox regression for overall survival and Recurrence-Free Survival to TNM.

	Overall Survival			Recurrence-Free Survival		
Stage	*f* *	*p* ***	HR ****	*f* **	*p* ***	HR ****
I	369			368		
II	109	<0.0001	2.27 (1.59–3.26)	109	<0.0001	2.14 (1.44–3.17)
III	105	<0.0001	4.43 (3.20–6.14)	104	<0.0001	3.48 (2.39–5.06)
IV	36	<0.0001	10.90 (7.17–16.57)	36	<0.0001	10.82 (6.36–18.41)

* *f*—Frequency: 69 patients did not have TNM, so all categorized of 622 in sum, and 4 patients did not have overall survival, so all markers will have 617 in sum; ** 74 patients not available to recurrence, so all categorized of 617 in sum; *** Significant (*p* < 0.05); **** HR (CI)—Hazard Ratio (confidence interval)—Log Rank (Mantel–Cox).

**Table 13:** Multivariate Cox regression for overall survival.

Variables	Characteristics	Freq *	*p* **	Hazard Ratio of Exp(B) ***
Hemoglobin Count (g/dL)	until 8	29	<0.0001	1.0
Leukocytes Count (leukocytes/mm^3^)	Leukocytosis	101	<0.0001	2.55 (1.89–3.44)
Platelets Count (platelets/mm^3^)	Thrombocytosis	50	<0.0001	2.46 (1.69–3.58)
Tumor Size ****	>4	192	<0.0001	4.32 (1.98–9.41)
Invasion ****	Yes	201	0.05	1.71 (1.12–2.62)
Neural Invasion ****	Yes	48	0.05	2.06 (1.14–3.71)
Depth of stromal invasion ****	Total	243	<0.0001	3.43 (1.94–6.06)
Compromised margin ****	Yes	45	<0.0001	2.98 (1.88–4.74)
Lymph node metastasis ****	Positive	125	<0.0001	2.81 (1.92–4.11)
TNM Pathological Stage ****	IV	34	<0.0001	7.58 (4.50–12.75)
External Radiation Therapy	Yes	393	<0.0001	1.68 (1.28–2.21)
Chemotherapy	Yes	294	<0.01	1.362 (1.06–1.74)

* Freq: Frequency; ** *p* Significant (*p* < 0.05); *** Log Rank (Mantel–Cox); **** The assessment was based on the 468 patients with cervical cancer who underwent surgery.

## Data Availability

The data that support the findings of this study are available from the corresponding author upon reasonable request.

## Supplementary Materials

The following supporting information can be downloaded at: https://www.mdpi.com/article/10.3390/cancers16142496/s1, Table S1: Specification, clone, dilution, antigenic recovery, and incubation time of the primary antibodies; Table S2: Positivity pattern of antibody cell expression; Table S3: Distribution of patients by lifestyle database, laboratory analysis, clinical stage, treatment, and clinicopathological; Table S4: Cox-regression for overall survival to lifestyle, laboratory analysis, of clinical stage, clinicopathological characteristic and treatment; Table S5: Immunoexpression pattern of VIM, E-cad, MMP9, SNAIL, TGFβ, CD25, FOXP3, MIF, IL-17, IL-10, PD-L1, Bcl-2, VEGFα, and Ki-67 and correlation of association with TNM.
