# Supplementary material for: High M2-TAM Infiltration and STAT3/NF-κB Signaling Pathway as a Predictive Factor for Tumor Progression and Death in Cervical Cancer
Source: Cancers (Basel). 2024 Jul 9;16(14):2496. doi: 10.3390/cancers16142496 (PMC11275153; doi:10.3390/cancers16142496)
Supplement: Supplementary file 1 [file cancers-16-02496-s001.zip › cancers-3069441-supplementary.pdf]

High M2-TAM infiltration and STAT3/NF-κB signaling pathway as a predictive factor for tumor progression and death in cervical cancer.

George Alexandre Lira MD, PhD[1,3,4,5], Fábio Medeiros de Azevedo MD, PhD[6], Ingrid Gabrielle dos Santos Lins MD[5], Isabelle de Lima Marques MD3, Giovanna Afonso Lira MD[5], Christina Eich PhD[4], Raimundo Fernandes de Araújo Júnior PhD [1,2,3,4]

[1] Cancer and Inflammation Research Laboratory, Department of Morphology, Federal University of Rio Grande do Norte Natal, RN 59072-970, Brazil
[2] Post-Graduation in Structural and Functional Biology, Federal University of Rio Grande do Norte, Natal, RN 59072-970, Brazil
[3] Post-Graduation in Health Science, Federal University of Rio Grande do Norte, Natal, RN 59072-970, Brazil
[4] Department of Radiology, Leiden University Medical Center, Leiden, ZA 2333, the Netherlands
[5] League Against Cancer from Rio Grande do Norte, Advanced Oncology Center, Natal, RN 59075-740, Brazil
[6] Pathology Department, Federal University of Rio Grande do Norte, Natal, RN 59012-570, Brazil

Conflict of Interest Statement:
The authors declare no potential conflicts of interest.

***Correspondence to:**
Prof. Raimundo Fernandes de Araujo Junior, University Campus, Federal University of Rio Grande do Norte, Morphology Department, Brazil. Phone: +558498934310 Email: fernandes.araujo@ufrn.br

Supplementary

Methods

2.1. *Tissue samples and data collection*

This research is a retrospective and longitudinal study of 1390 women diagnosed with cervical cancer from 2003 to 2017 registered at the archives of the Advanced Oncology Center at the League Against Cancer from Rio Grande do Norte (LRNCC), Natal, Brazil. After the exclusion of 644 patients from the study due to insufficient follow-up or missing data or insufficient pathological material (Fig 1), paraffin blocks and medical records from 746 women were collected for clinical-histopathological analysis. However, 55 women were excluded for having been diagnosed with cervical intraepithelial neoplasia (benignity). Previously, 691 cases had histopathological confirmation by a surgical pathologist as cervical cancer according to histology types: Squamous cell carcinoma, Adenocarcinoma and Adenosquamous. Likewise, the inclusion criteria adopted to select 691 cases were as follows: (I) patients underwent surgery with definitive histopathologic diagnosis of cervical cancer, (II) scored by TNM and International Federation of Gynecology and Obstetrics (FIGO) staging system of cervical cancer based on the Union for International Cancer Control (UICC) and FIGO guidelines eighth edition (22), (III) patients submitted to preoperative chemotherapy or radiotherapy treatment, (IV) with no surgical contraindication that could affect prognosis and (V) with surgical material enough to performed the tissue microarray (TMA). Furthermore, sociodemographic and clinicopathological data were also collected (Supplementary). The Institutional Review Board (IRB) from LRNCC, (CAE 2077017-2644266-2761333-3071847, Natal, Brazil) approved this study.

2.1.1 *Sociodemographic and clinicopathological data*

Sociodemographic, clinical health, blood analysis, histopathological and therapeutic data were obtained from individual patient records. In this context, collected data related to lifestyle, tobacco smoking and alcohol consumption, and patient age at the diagnosis time. Furthermore, histopathology characteristics of tumors were highlighted, such as grade of cellular differentiation, angiolymphatic invasion, neural invasion, depth of stromal invasion, involvement of the tumor margin, lymph node metastasis from pelvic and retroperitoneum regions, histology type of cervical cancer, FIGO and TNM stages. Tumor recurrence after treatment, death, number of hemoglobin, leukocytes, and platelets also were annotated.

2.3 Quantitative Evaluation of Immunostaining

Slides were analyzed using Aperio CS2 device (Aperio, Vista, CA, USA) and Image Scope software (Aperio, Vista, CA, USA). Representative areas of each sample were analyzed by electronic morphometry. Necrotic and autolytic areas and loss of areas greater than 10% were discarded to analyze. According to the staining intensity, it was considered positive, with cells-stained brown in the nucleus, and/or cytoplasm, and/or membrane (Table S2). The expression of markers was considered according to the numerical result of the software image analysis algorithms which is based on the RGB color system from a scale of 0 to 255 pixels: for the nuclear positivity pattern, the algorithm used was “Nuclear v9”, for the cytoplasm, the algorithm used was “Positive Pixel Count v9” and for membrane, the algorithm used was “Membrane v9” (24,25). In this line, based on the numerical result of the parameter in pixel, a median between the color intensity and the number of cells with positive staining was obtained and considered as “negative”, “weak” (<5%), “moderate” (5-50%) and “strong” (>50%) when, and negative for unmarked ones. Regarding Ki-67, the quantification of Ki-67 positive cells within the hotspot was calculated by the percentage of positive cells in a fixed area of 0.05mm2 of the sample (26-28). A hotspot was subjectively defined as the area with the highest density of Ki-67-positive tumor cells identified at 20× magnification compared to areas of surrounding tumor tissue and then at 40× magnification. The mean percentage of Ki-67-positive tumor cells, relative to all tumor cells within the hotspot, was estimated by the percentage of Aperio-positive nuclei. To classify, we categorize Ki-67 into distinct low- and high-proliferating tumors, including up to 15% and greater than 15%, respectively (29).

A total of 691 patients who underwent immunohistochemical marking were divided into two groups: negative or weak for categorization "1", and moderate or strong markings were categorized as "2" for the positive group. Ki-67 was categorized as either 1 for low proliferation or 2 for high proliferation.

2.4. Follow-up

In all 691 women with cervical cancer followed up from 2003 to 2017, 468 of them had surgery with a histopathological investigation of HPV, angiolymphatic invasion, neural invasion, depth of stromal invasion, involvement of the tumor margin and lymph node metastasis from pelvic and retroperitoneum regions. 295 cases had chemotherapy, 394 cases

had external radiotherapy, 339 cases had internal radiotherapy and 149 cases had postsurgical radiotherapy combined with chemotherapy. Overall survival (OS) was calculated as the length of time from the date of diagnosis or the start of treatment for a disease, such as cancer, that patients diagnosed with the disease are still alive (30). Recurrence-free survival (RFS) was calculated as the measure of time from random assignment to cancer recurrence or death from any cause (31).

Supplementary Tables

Supplementary Table S1. Specification, clone, dilution, antigenic recovery, and incubation time of the primary antibodies.

Antibody	Manufacturer	Clone	Dilution	Antigen retrieval	Incubation
Anti-Vimentin	Flex dako – Denmark	Monoclonal mouse –v9	Flex	Trilogy, 1:100, pascal, 30 min	60 minutes
Anti-NF-κB	Santa Cruz Biotech P65(a) sc-109 - lot 1007	Polyclonal - IgG	1:400	Trilogy, 1:100, pascal, 30 min	Overnight
Anti-TGFß1	Santa Cruz Biotech (V): sc-146-lot G0114	Polyclonal – IgG Rabbit	1:200	Trilogy, 1:100, pascal, 30 min	Overnight
Anti-PD-L1	Bioisb inc Cd274	Rbt – pdl1 Bsb 2652	1:100	Trilogy, 1:100, pascal, 30 min	60 minutes
Anti-E-cadherin	Flex dako Nch- 38	Monoclonal Antihuman	Flex	Trilogy, 1:100, pascal, 30 min	60 minutes
Anti-MIF	Santa Cruz Biotech Fl-115 sc-20121	Polyclonal Rabbit	1:200	Trilogy, 1:100, pascal, 30 min	60 minutes
Anti-VEGFα	Boster Biological Tech Pa1080	100 ug - Lot n010 Human-mouse-rat	1:500	Trilogy, 1:100, pascal, 30 minutes	Overnight
Anti-CD25	Invitrogen Ma51268	Anticorpo Il2r1	1:200	Trilogy, 1:100, pascal, 30 minutes	Overnight
Anti-CD163	Proteintech 16646-1-AP	Rabbit PolyAb	1:200	Trilogy, 1:100, pascal, 30 minutes	60 minutes
Anti-IL-10	Santa Cruz Biotech Sc-1783	M18 cabra Polyclonal	1:200	Trilogy, 1:100, pascal, 30 minutes	Overnight

Anti-Ki-67	Dbs Sp6	Monoclonal antiki67 rabbit	1:200	Trilogy, 1:100, pascal, 30 minutes	60 minutes
Anti-STAT3	Santa Cruz biotech F-2 -Sc1783	Monoclonal mouse igg1k	1:200	Trilogy, 1:100, pascal, 30 minutes	Overnight
Anti-SNAIL	Santa Cruz Biotech Sc271977	G6	1:250	Trilogy, 1:100, pascal, 30 minutes	Overnight
Anti-CD204	Invitrogen J5htr3	Antihhuman Cd204	1:200	Trilogy, 1:100, pascal, 30 minutes	Overnight
Anti-FOXP3	Santa Cruz Biotech (2A11G9): Sc-53876	Antihuman	1:100	Trilogy, 1:100, pascal, 30 minutes	Overnight
Anti-MMP9	Boster Pb10008	Polyclonal	1:100	Trilogy, 1:100, pascal, 30 minutes	Overnight
Anti-Bcl-2	Dako	124	1:200	Trilogy, 1:100, pascal, 30 minutes	60 minutes
Anti-IL-17	Santa Cruz Biotech Sc7927 (h132)	Polyclonal	1:200	Trilogy, 1:100, pascal, 30 minutes	Overnight

Supplementary Table S2. Positivity pattern of antibody cell expression.

Antibody	Cellular Expression Pattern	Iavg maximum	Iavg minimum	Interval (weak)	Interval (moderate)	Interval (strong)
Anti-Vimentin	Cytoplasmic / Membrane	184.3	54.4	184.3 – 177.8	177.8 – 119.4	119.4 – 54.4
Anti-NF-κB	Cytoplasmic	186.1	61.78	186.1 – 179.9	179.9 – 123.9	123.9 – 61.78
Anti-TGFß1	Cytoplasmic	173.95	99.94	173.95 – 170.24	170.24 – 136.94	136.94 – 99.94
Anti-PD-L1	Cytoplasmic	183.61	113.02	183.61 – 180.08	180.08 – 148.42	148.32 – 113.02
Anti-E-cadherin	Cytoplasmic / Membrane	206.3	55.71	206.3 – 198.77	198.77 – 131.00	131.00 – 55.71
Anti-MIF	Cytoplasmic	163.98	51.03	163.98 – 158.33	158.33 – 107.50	107.50 – 51.03
Anti-VEGFα	Cytoplasmic	155.34	56.85	155.34 – 150.42	150.42 – 106.10	106.10 – 56.85
Anti-CD25	Cytoplasmic	180	84.3	180 – 175	175 – 132	132 – 84.3
Anti-CD163	Cytoplasmic	211	89.52	221 – 202.92	204.92 – 150.26	150.26 – 89.52
Anti-IL-10	Cytoplasmic	186	103	186 – 181	181 – 144	144 - 103
Anti-STAT3	Cytoplasmic / Nuclear	198	36	198 – 189.99	189.99 – 117	117 - 36
Anti-SNAIL	Nuclear	207.00	107.00	207.00 – 202.00	202.00 – 157.00	157.00 – 107.00
Anti-CD204	Cytoplasmic / Nuclear	152.2	86.2	152.2 – 148.92	148.92 – 119.21	119.21 – 86.2

Anti-FOXP3	Cytoplasmic	204.8	87.57	204.8 – 198.9	198.9 – 146.2	146.2 – 87.57
Anti-MMP9	Cytoplasmic / Nuclear	170.25	49.34	170.25 – 164.2	164.2 – 109.8	109.8 – 49.34
Anti-Bcl-2	Cytoplasmic / Nuclear	182	72.34	182.00 – 176.51	176.51 – 127.17	127.17 – 72.34
Anti-IL-17	Cytoplasmic	163.5	82.27	163.5 – 159.43	159.43 – 122.88	122.88 – 82.27
Anti-Ki-67*	Nuclear	1	96	*Analyzed by percent positive nuclei in hotspot (%)		

Supplementary Table S3. Distribution of patients by lifestyle database, laboratory analysis, clinical stage, treatment, and clinicopathological.

Variable	Characteristics	Frequency	Percentage
Tobacco Smoking	Smoker	227	32.9%
	No-smoker	292	42.3%
	Not available	172	24.9%
	Total Number	691	100.0%
Alcohol consumption	Alcoholic	97	14.0%
	No-alcoholic	383	55.4%
	Not available	211	30.5%
	Total	691	100.0%
Hemoglobin count (g/dL)	Above 12	307	44.4%
	> 10 e ≤ 12	150	21.7%
	> 8 e ≤ 10	74	10.7%
	Until 8	31	4.5%
	Not available	129	18.7%
	Total	691	100.0%
Leukocytes count (leukocytes / mm^3)	Leukocytosis	103	14.9%
	Normal	427	61.8%
	Leukopenia	26	3.8%
	Not available	135	19.5%
	Total	691	100.0%
Platelets count (platelets / mm^3)	Thrombocytosis	52	7.5%
	Normal	479	69.3%
	Thrombocytopenia	8	1.2%
	Not available	152	22.0%
	Total	691	100.0%
Treatment of surgery	No	223	32.28%
	Yes	468	67.72%
	Total Number	691	100.0%
Tumor Size after Surgery*	< 2	71	15.17%
	2 to 4	139	29.70%
	> 4	192	41.10%
	Not available	66	41.8%
	Total	468	100.0%
Histological type	Squamous cell carcinoma	480	69.5%

	Adenocarcinoma	109	15.8%
	Adenosquamous	77	11.1%
	Not available	25	3.7%
	Total	691	100.0%
HPV (koilocyte)	No	384	55.6%
	Yes	179	25.9%
	Not available	128	18.5%
	Total	691	100.0%
Angiolymphatic Invasion*	No	169	36.1%
	Yes	203	43.4%
	Not available	96	20.5%
	Total	468	100.0%
Neural Invasion*	No	139	29.7%
	Yes	49	10.50%
	Not available	280	59.80%
	Total	468	100.0%
Degree of pathological differentiation	Well differentiated	37	5.4%
	Moderately differentiated	168	24.3%
	Little differentiated / Undifferentiated	56	8.1%
	Not	430	62.2%
	Total	691	100.0%
Depth of stromal invasion*	1/3 internal	104	22.2%
	2/3 internal	61	13.0%
	Total	242	51.7%
	Not available	61	13.0%
	Total	468	100.0%
Compromised margin*	No	401	85.7%
	Yes	45	9.6%
	Not available	22	4.7%
	Total	468	100.0%
Lymph node metastasis*	Negative	317	67.7%
	Positive	125	26.7%
	Not available	26	5.6%
	Total	468	100.0%
Number of resected retroperitoneal lymph nodes*	0	120	25.64%
	1 a 3	236	50.42%
	> 3	100	21.36%
	Not available	12	0.02%
	Total	468	100.0%
Number of resected obturators pelvic lymph nodes*	0	37	7.90%
	1 a 5	137	29.27%
	6 a 10	211	45.08%
	> 10	72	15.38%
	Not available	11	2.35%
	Total	468	100.0%
Number of resected iliac pelvic lymph nodes*	0	22	4.70%
	1 a 5	134	28.63%
	6 a 10	216	46.15%
	> 10	84	17.94%

	Not available	12	0.02%
	Total	468	100.0%
TNM Pathological Staging*	I	275	58.8%
	II	38	8.1%
	III	108	23.1%
	IV	34	7.3%
	Not available	13	2.8%
	Total	468	100.0%
External Radiation Therapy	No	291	42.1%
	Yes	394	57.0%
	Not available	6	0.9%
	Total	691	100.0%
Internal Radiation Therapy	No	344	49.8%
	Yes	339	49.1%
	Not available	8	1.2%
	Total	691	100.0%
Chemotherapy	No	388	56.2%
	Yes	295	42.7%
	Not available	8	1.2%
	Total	691	100.0%

*The assessment was based on the 468 patients with cervical cancer who underwent surgery

Supplementary Table S4. Cox-regression for overall survival to lifestyle, laboratory analysis, of clinical stage, clinicopathological characteristic and treatment.

Variable	Characteristics	Freq*	*p***	Hazard ratio of Exp(B)***
Tobacco Smoking	No	290		
	Yes	225	0.18	1.20 (0.91 – 1.58)
Alcohol consumption	No	382		
	Yes	95	0.36	0.85 (0.59 – 1.21)
Hemoglobin Count (g/dL)	until 8	29	***< 0.0001***	
	> 8 e ≤ 10	74	***0.001***	0.42 (0.25 – 0.71)
	> 10 e ≤ 12	149	***< 0.0001***	0.22 (0.13 – 0.37)
	> 12	306	***< 0.0001***	0.13 (0.08 – 0.22)
Leukocytes Count (leukocytes/ mm^3)	Normal	425	***< 0.0001***	
	Leukocytosis	101	***< 0.0001***	2.55 (1.89 – 3.44)
	Leukopenia	26	0.318	0.65 (0.29 – 1.49)
Platelets Count (platelets/ mm^3)	Normal	477	***< 0.0001***	
	Thrombocytopenia	8	0.206	1.77 (0.72 – 4.33)
	Thrombocytosis	50	***< 0.0001***	2.46 (1.69 – 3.58)
Tumor size after Surgery (cm) ****	< 2	70	***<0.0001***	
	2 a 4	139	0.08	2.07 (0.91 – 4.74)
	> 4	192	***<0.0001***	4.32 (1.98 – 9.41)
Histological type	Squamous cell carcinoma	478	0.33	
	Adenocarcinoma	108	0.25	0.81 (0.57 – 1.16)

	Adenosquamous	76	0.35	1.19 (0.81 – 1.73)
HPV (koilocyte)	No	381		
	Yes	178	***<0.0001***	0.45 (0.31 – 0.63)
Angiolymphatic Invasion ****	No	169		
	Yes	201	***0.05***	1.71 (1.12 – 2.62)
Neural Invasion ****	No	140		
	Yes	48	***0.05***	2.06 (1.14 – 3.71)
Depth of stromal invasion ****	1/3 internal	103	***<0.0001***	
	2/3 internal	61	0.38	0.65 (0.25 – 1.70)
	Total	243	***<0.0001***	3.43 (1.94 – 6.06)
Compromised margin ****	No	400		
	Yes	45	***<0.0001***	2.98 (1.88 – 4.74)
Lymph node metastasis ****	Negative	315		
	Positive	125	***<0.0001***	2.81 (1.92 – 4.11)
Number of resected retroperitoneal lymph Nodes****	0	120	***0.01***	
	1 a 3	234	0.69	0.91 (0.56 – 1.46)
	> 3	100	***<0.05***	1.68 (1.02 – 2.77)
Number of resected obturator pelvic lymph Nodes ****	0	37	0.59	
	1 a 5	137	0.62	0.83 (0.41 – 1.70)
	6 a 10	209	0.44	0.76 (0.38 – 1.51)
	>10	72	0.88	1.05 (0.50 – 2.22)
Number of resected iliac pelvic lymph nodes ****	0	22	0.07	
	1 a 5	133	0.10	0.51 (0.22 – 1.16)
	6 a 10	215	***0.05***	0.46 (0.20 – 1.02)
	>10	84	***0.50***	0.75 (0.33 – 1.72)
TNM Pathological Stage ****	I	274	***<0.0001***	
	II	37	***<0.0001***	2.6 (1.41 – 4.97)
	III	110	***<0.0001***	2.8 (1.78 - 4.40)
	IV	34	***<0.0001***	7.58 (4.50 – 12.75)
External Radiation Therapy	No	288		
	Yes	393	***<0.0001***	1.68 (1.28 – 2.21)
Internal Radiation Therapy	No	341		
	Yes	338	***<0.01***	0.71 (0.55 – 0.91)
Chemotherapy	No	385		
	Yes	294	***<0.01***	1.36 (1.06 – 1.74)

*Freq: Frequency
***p* Significant (p<0.05)
***Log Rank (Mantel-Cox)
****The assessment was based on the 468 patients with cervical cancer who underwent surgery

Supplementary Table S5. Immunoexpression pattern of VIM, E-cad, MMP9, SNAIL, TGFβ, CD25, FOXP3, MIF, IL-17, IL-10, PD-L1, Bcl-2, VEGFα, and Ki-67 and correlation of association with TNM.

		TNM					
Marker		I	II	III	IV	Total	*p**
VIM	1	43 (6.9%)	18 (2.9%)	31 (5.0%)	7 (1.1%)	99 (15.9%)	
	2	327 (52.6%)	91 (14.6%)	75 (12.1%)	30 (4.8%)	523 (84.1%)	***< 0.001***
E-cad	1	136 (21.9%)	56 (9.0%)	75 (12.1%)	20 (3.2%)	287 (46.1%)	
	2	234 (37.6%)	53 (8.5%)	31 (5.0%)	17 (2.7%)	335 (53.9%)	***< 0.0001***
MMP9	1	42 (6.8%)	9 (1.4%)	14 (2.3%)	2 (0.3%)	67 (10.8%)	
	2	328 (59.0%)	100 (18.0%)	92 (16.5%)	35 (6.3%)	555 (89.2%)	0.50
SNAIL	1	154 (24.8%)	26 (4.2%)	24 (3.9%)	4 (0.6%)	208 (33.4%)	
	2	216 (34.7%)	83 (13.3%)	82 (13.2%)	33 (5.3%)	414 (66.6%)	***< 0.0001***
TGFβ	1	285 (45.8%)	81 (13.0%)	73 (11.7%)	26 (4.2%)	465 (74.8%)	
	2	85 (13.7%)	28 (4.5%)	33 (5.3%)	11 (1.8%)	157 (25.2%)	0.32
CD25	1	142 (22.8%)	60 (9.6%)	58 (9.3%)	21 (3.4%)	281 (45.2%)	
	2	228 (36.7%)	49 (7.9%)	48 (7.7%)	16 (2.6%)	341 (54.8%)	***< 0.001***
FOXP3	1	89 (14.3%)	17 (2.7%)	28 (4.5%)	10 (1.6%)	144 (23.2%)	
	2	281 (58.7%)	92 (19.2%)	78 (16.3%)	27 (5.6%)	478 (76.8%)	0.18
MIF	1	41 (6.6%)	16 (2.6%)	31 (5.0%)	7 (1.1%)	95 (15.3%)	
	2	329 (52.9%)	93 (15.0%)	75 (12.1%)	30 (4.8%)	527 (84.7%)	***0.0001***
IL-17	1	46 (7.4%)	11 (1.8%)	23 (3.7%)	5 (0.8%)	85 (13.7%)	
	2	324 (52.1%)	98 (15.8%)	83 (13.3%)	32 (5.1%)	537 (86.3%)	0.07
IL-10	1	337 (54.2%)	100 (16.1%)	102 (16.2%)	36 (5.8%)	575 (92.4%)	
	2	33 (5.3%)	9 (1.4%)	4 (0.6%)	1 (0.2%)	47 (7.6%)	0.24
PD-L1	1	321 (51.6%)	102 (16.4%)	101 (16.2%)	37 (5.9%)	561 (90.2%)	
	2	49 (7.9%)	7 (1.1%)	5 (0.8%)	0 (0.0%)	61(9.8%)	***< 0.01***
Bcl-2	1	83 (13.3%)	17 (2.7%)	30 (4.8%)	9 (1.4%)	139 (22.3%)	
	2	287 (46.1%)	92 (14.8%)	76 (12.2%)	28 (4.5%)	483 (77.7%)	0.15
VEGFα	1	60 (9.6%)	32 (5.1%)	34 (5.5%)	12 (1.9%)	138 (22.5%)	
	2	310 (49.8%)	77 (12.4%)	72 (11.6)	25 (4.0%)	484 (77.8%)	***< 0.001***
Ki-67	1	168 (27.0%)	24 (3.9%)	17 (2.7%)	4 (0.6%)	213 (34.4%)	
	2	202 (32.5%)	85 (13.7%)	89 (14.3%)	33 (5.3%)	409 (65.8)	***< 0.0001***

* Fisher's exact test (significant p<0.05)
69 patients didn't have TNM, so all categorized of 622 in sum.
